# Gender and mental health service use in bipolar disorder: national cohort study

**DOI:** 10.1192/bjo.2020.117

**Published:** 2020-11-06

**Authors:** Ruth Cunningham, Marie Crowe, James Stanley, Tracy Haitana, Suzanne Pitama, Richard Porter, Jo Baxter, Tania Huria, Roger Mulder, Mau Te Rangimarie Clark, Cameron Lacey

**Affiliations:** Department of Public Health, University of Otago, New Zealand; Department of Psychological Medicine, University of Otago, New Zealand; Department of Public Health, University of Otago, New Zealand; Māori/Indigenous Health Institute (MIHI), University of Otago, New Zealand; Māori/Indigenous Health Institute (MIHI), University of Otago, New Zealand; Department of Psychological Medicine, University of Otago, New Zealand; Kōhatu Centre for Hauora Māori, Dunedin School of Medicine, University of Otago, New Zealand; Māori/Indigenous Health Institute (MIHI), University of Otago, New Zealand; Department of Psychological Medicine, University of Otago, New Zealand; Māori/Indigenous Health Institute (MIHI), University of Otago, New Zealand; Department of Psychological Medicine, University of Otago, New Zealand; and Māori/Indigenous Health Institute (MIHI), University of Otago, New Zealand

**Keywords:** Bipolar disorder, epidemiology, gender, service use

## Abstract

**Background:**

Despite evidence of gender differences in bipolar disorder characteristics and comorbidity, there is little research on the differences in treatment and service use between men and women with bipolar disorder.

**Aims:**

To use routine data to describe specialist mental health service contact for bipolar disorder, including in-patient, community and support service contacts; to compare clinical characteristics and mental health service use between men and women in contact with secondary services for bipolar disorder.

**Method:**

Cross-sectional analysis of mental health patients with bipolar disorder in New Zealand, based on complete national routine health data.

**Results:**

A total of 3639 individuals were in contact with specialist mental health services with a current diagnosis of bipolar disorder in 2015. Of these 58% were women and 46% were aged 45 and over. The 1-year prevalence rate of bipolar disorder leading to contact with specialist mental health services was 1.56 (95% CI 1.50–1.63) per 100 000 women and 1.20 (95% CI 1.14–1.26) per 100 000 men. Rates of bipolar disorder leading to service contact were 30% higher in women than men (rate ratio 1.30, 95% CI 1.22–1.39). The majority (68%) had a diagnosis of bipolar I disorder. Women were more likely to receive only out-patient treatment and have comorbid anxiety whereas more men had substance use disorder, were convicted for crimes when unwell, received compulsory treatment orders and received in-patient treatment.

**Conclusions:**

Although the prevalence of bipolar disorder is equal between men and women in the population, women were more likely to have contact with specialist services for bipolar disorder but had a lower intensity of service interaction.

## Background

Bipolar disorder is a recurrent, disabling mental health condition affecting 1–2.5% of the population over their lifetime.^1^ The lifetime prevalence estimates for this disorder in the USA are 1.0% for bipolar I disorder; 1.1% for bipolar II disorder and 2.4% for subthreshold bipolar disorder.^2^ In New Zealand the 2004 national representative mental health survey Te Rau Hinengaro^3^ reported a 12-month prevalence of bipolar I and II disorders (combined) of 1%, and lifetime prevalence of bipolar including subthreshold disorder at 3.8% (4.1% in men and 3.6% in women).

## Impact of bipolar disorder

Bipolar disorder is one of the world's ten most disabling conditions, associated with substantial morbidity and mortality.^4–6^ It is responsible for the loss of more disability-adjusted life-years than all forms of cancer or major neurological conditions such as epilepsy and Alzheimer's disease,^7^ because of its early onset, and chronicity across the lifespan, as well as the severity of functional impairment and the impact on quality of life.^8^ An 11-country study of the prevalence and correlates of bipolar disorder^7^ found that 75% of those with bipolar disorder reported severe levels of depressive symptoms and a comparable magnitude of severe role impairment. Depressive morbidity in patients with bipolar disorder accounts for 86% of the time ill and even patients with bipolar disorder receiving treatment are ill for about 40% of the time.^9^ The risk of suicide in bipolar disorder is 20 to 30 times that in the general population.^10^ Consequentially people with bipolar disorder have high rates of specialist mental health service use (in-patient and community based) particularly during acute episodes.

## Gender differences and comorbidities

Most studies have found similar prevalence of bipolar disorder among men and women^3^ and no clear evidence of any gender differences in age at onset or polarity at onset.^11^ Although prevalences are similar, there are important differences in comorbidities between men and women. Comorbid bulimia nervosa, anxiety disorders, post-traumatic stress disorder,^12^ as well as medical disorders including migraine^13^ hypothyroidism and inflammatory disorders^14^ are more commonly diagnosed in women with bipolar disorder, and suicide attempts^15^ are also more common. The impact of hormonal and reproductive factors are well documented.^11,16^ There is evidence of higher rates of comorbid substance use disorders,^17^ criminal activity^12^ and completed suicide in men.^10^ Differential treatment in both clinical and justice services may influence reported gender differences.^18^

## Mental health services

The majority of specialist mental health treatment is now provided in the community including in New Zealand where in 2017 92% of those receiving specialist mental health treatment were not treated as in-patients in hospital.^19^ Despite this, much of the research on psychiatric treatment has focused on in-patients. The collection and analysis of routine data now allows for the examination of whole-of-nation service-use patterns. It is therefore possible to explore the extent to which hospital admissions, community treatment and compulsory treatment are being used in managing certain categories of mental illness, including bipolar disorder, using a complete population data-set.

There is relatively little research on the differences in treatment and service use between men and women with bipolar disorder, but a small number of studies suggest differences in treatment. A recent study from Austria^20^ reported that women had higher rates of hospital admissions for bipolar disorder than men, despite a similar population prevalence. A Danish study identified that women with bipolar disorder were more likely to be treated as out-patients (rather than in-patients) in their first contact with services than men, but when they were treated as in-patients, this was for longer periods.^17^ It is not clear why this may be the case. Women do appear to have longer episodes of bipolar depression, which is a possible explanation.^21,22^ Another study from Sweden found differences in prescribing and other treatments between men and women with bipolar disorder that were not explained by clinical differences.^23^

## Aims

This study uses whole-of-nation routine clinical data on the patient population in contact with specialist mental health services for bipolar disorder in New Zealand to explore gender differences in treatment.

Our aims were:

- to use routine data to describe specialist mental health service contact for bipolar disorder, including in-patient and community treatment and non-clinical support service provision;
- to compare clinical characteristics and mental health service use between men and women in contact with specialist mental health services for bipolar disorder.

## Method

### Study design and setting

This was a cross-sectional analysis of mental health patients with bipolar disorder in New Zealand, based on complete national-level routine clinical data collated in the Programme for Integrated Mental Health Data (PRIMHD).

### Population

All living individuals with an open primary or provisional diagnosis of bipolar disorder (ICD-10-AM^24^ F31; or DSM-IV^25^ 296.00, 296.4X, 296.6, 296.5, 296.7, 296.80, 296.89) as recorded in the PRIMHD data source by national specialist mental health services in 2015 (i.e. either a diagnosis with a recorded start date prior to 1 January 2015 and an end date after 1 January 2015, or a diagnosis starting during 2015). Public specialist mental health services in New Zealand are provided to the roughly 4% of the general population with the highest and most acute mental health need. There is also a small amount of private specialist care but expense and low health insurance coverage make this minimal, and so public services provide an almost complete picture of those with high mental health need.

Mental health services for those aged 65 and older are not provided or funded in a consistent way in New Zealand across District Health Boards (the main organisational level of public healthcare delivery), resulting in large numbers of missing patients in this age group in the national PRIMHD collection.^26^ The present study is thus limited to adults aged 18–64 at mid-year (1 July 2015).

### Data sources

PRIMHD is a centrally managed national clinical data-set maintained by the Ministry of Health, to which all publicly funded secondary mental health service providers report their service provision.^27^ For this project, de-identified records for demographic, clinical and service-use characteristics were requested for all those with an open (current) bipolar disorder diagnosis between 2009 and 2015. Data from 2015 are presented in this paper as the most recent available snapshot of this group of people with bipolar disorder.

Additional data were sourced from the wider Ministry of Health National Collections data (including demographics, and information on all hospital admissions including mental and physical health admissions).^28^ All records were linked by encrypted National Health Index number (NHI), a unique identifier for people engaging with the New Zealand public healthcare system.

Population denominator data were drawn from the New Zealand Census. Population counts for 2015 were calculated by age and gender groups by linear extrapolation of changes in population counts between the 2006 and 2013 censuses.

### Consent and ethics statement

All data sources were deidentified and informed consent was not required under New Zealand Health Information Privacy Code 1994. The authors assert that all procedures contributing to this work comply with the ethical standards of the relevant national and institutional committees on human experimentation and with the Helsinki Declaration of 1975, as revised in 2008. All procedures involving human patients were approved by New Zealand Health and Disability Ethics Committee (ref: 16/STH/137)

### Variables

Gender is recorded on the health service data records for each person in contact with the New Zealand health system. Although the data collection system allows for an ‘other’ option, all of those with an open diagnosis of bipolar disorder in 2015 had a male or female gender recorded. Age was calculated at the midpoint of the study year (1 July 2015) based on date of birth in the master NHI table. Descriptive statistics and age-standardised rates were based on three age groups (18–29, 30–44, 45–64 years).

Self-reported ethnic group is recorded on health service data (in the master NHI table); individuals have the opportunity to update their ethnicity record at each health service contact. Up to three ethnic identities can be recorded for an individual in the master NHI data source. The analysis presented here reports descriptive statistics for ethnicity for Māori (including all those who had Māori ethnicity recorded with or without other ethnic identities) and non-Māori (all other) patients.^29^ Information on socioeconomic deprivation was classified using NZDep2013,^30^ an area-based measure of deprivation based on 2013 New Zealand Census data (defined at the Census area unit level, and assigned for each individual), and presented in quintiles (1, least deprived). Missing NZDep values are enumerated in the results, and are usually the result of either absent/invalid address data in the National Collections records (which may be because of homelessness) or having an address that was not available/valid at the time the NZDep index was created from the 2013 Census.

### Clinical variables

In New Zealand, for in-patient admissions, coding of diagnosis is carried out by trained coders who code the diagnoses based on a set of rules applied to the clinical file. For out-patient care, at discharge or after 3 months of care, a diagnosis must be entered by clinicians. The system does allow the likelihood that diagnoses can be added, particularly by clinicians without necessarily taking into account previous diagnoses, for example an episode of care may be diagnosed as ‘psychosis’ while the previous diagnosis has been bipolar disorder. The system of coding is similar to that in Australia.

Available psychiatric diagnoses (both provisional and confirmed) were used if they were open (current) in 2015 (i.e. the recorded start date for the diagnosis was prior to 31 December 2015 and the recorded end date for the diagnosis was after 1 January 2015). bipolar disorder diagnosis was categorised into bipolar I disorder multiple episodes (ICD-10: F31.0-F31.7; DSM-IV: 2964–67), bipolar I disorder single episode (DSM-IV: 2960), bipolar II disorder (ICD-10: F31.81; DSM-IV: 29689) and bipolar disorder-NOS (not otherwise specified) (ICD-10: F31.9; DSM-IV: 29680).^31^ Where an individual had multiple bipolar disorder diagnoses recorded, a single diagnosis is reported based on the above prioritised order.

Comorbid psychiatric diagnoses recorded in PRIMHD (recorded as current during the study year) were categorised into other psychoses, alcohol use disorders, substance use disorder, other mood disorder, anxiety and related disorders, and personality disorder, with multiple comorbid diagnoses per individual permitted (see Supplementary Table 1 available at https://doi.org/10.1192/bjo.2020.117).

### Service-use variables

Individual service-use events (‘Activities’ in the PRIMHD data structure) were classified prior to the start of analysis in order to describe the range of service activities for the study population. These were broadly grouped into in-patient hospital admissions, community treatment activities and support service provision. Community treatment included face-to-face treatment by psychiatric services in out-patient clinics, emergency department and community settings. Treatment by forensic services, substance abuse services and crisis treatment contacts were also examined separately. Support service contacts included all non-clinical face-to-face patient contacts such as vocational support, peer support, cultural services, day activity programmes and other community support activities. Service-use events refer to a face-to-face contact on a single day, but it is not possible in the data to distinguish types of treatment events (for example community mental health nursing visit for depot injection versus psychiatric review versus psychological therapy session).

Legal status records were also examined to count the number of patients who were either already under (at start of year) or placed under (during the study year) any of the following groupings derived from the legal sections of the New Zealand Mental Health (Compulsory Assessment and Treatment) Act 1992: an assessment order (Section 11, 13 or 15), an in-patient treatment order (Section 29(3a) or 30) and a community treatment order (CTO, Section 29).

### Sample size

The study covers all patients with an open diagnosis of bipolar disorder in the 2015 calendar year, and precision of the estimates as driven by the attained sample size is encapsulated in the reported confidence intervals.

### Analysis

Descriptive statistics are provided for sociodemographic characteristics of the study population (with frequencies and percentages), stratified by gender and overall. Differences in these profiles by gender are summarised with a *P*-value from χ^2^ tests.

Rates of service use in 2015 are presented stratified by gender. The proportion of people with a current bipolar disorder diagnosis who had any contact with each type of service in 2015 are presented (with 95% CIs). Mean numbers of use for each service type (mean rate of activity count per person per annum, with 95% CI) are also presented to show the mean number of contacts with each service in the 2015 calendar year. Rate ratios (RRs) are presented for differences by gender, expressed as the ratio for women relative to men (with 95% CI). Population rates were calculated based on the 2013 New Zealand Census population stratified by gender.

Data processing for PRIMHD records was conducted in SAS 9.4 (SAS Institute, Cary, North Carolina, USA) and data analysis was completed using R 3.5 (R Institute, Vienna) and Excel (Microsoft Corporation, Redmond, Washington, USA).

## Results

A total of 3639 individuals aged 18 to 64 were identified from specialist service records with a current diagnosis of bipolar disorder during 2015. Table 1 shows the demographic and clinical characteristics. Women made up 58% of those accessing specialist services for bipolar disorder. Men and women had broadly similar distributions of age, ethnicity and deprivation (NZDep quintile) (Table 1), with 46% aged 45–64 and 22% of Māori ethnicity.

**Table 1:** Demographic and clinical characteristics of participants by gender

Variable	Women		Men		*P*^a^
	*n*	%	*n*	%	
Total^b^	2121	58.3	1518	41.7	
Ethnicity					
Māori	454	21.4	334	22.0	0.696
Non-Māori	1667	78.6	1184	78.0	
Age (mid-year), years^c^					
18–29	424	20.0	338	22.3	0.269
30–44	680	32.1	451	29.7	
45–64	987	46.5	705	46.4	
NZDep_Quintile^d^					
1	277	13.1	193	12.7	0.324
2	276	13.0	222	14.6	
3	396	18.7	251	16.5	
4	594	28.0	419	27.6	
5	571	26.9	429	28.3	
Bipolar diagnosis					
Bipolar I disorder	1282	60.4	968	63.8	<0.001
Bipolar I single episode	135	6.4	104	6.9	
Bipolar II disorder	303	14.3	145	9.6	
Bipolar NOS	401	18.9	301	19.8	
Psychiatric comorbidities					
No psychiatric comorbidity	1411	66.5	954	62.8	0.024
Psychoses	150	7.1	168	11.1	<0.001
Alcohol disorders	116	5.5	139	9.2	<0.001
Substance use disorders	131	6.2	182	12.0	<0.001
Mood disorders	156	7.4	108	7.1	0.833
Anxiety and related	228	10.7	99	6.5	<0.001
Personality disorder	124	5.8	66	4.3	0.054
Other recorded	67	3.2	32	2.1	0.069

NOS, not otherwise specified.

a. *P*-value from χ^2^ test comparing profile of each characteristic for men and women.

b. Row percentages (proportion of total sample by gender).

c. 54 people were aged under 18 at mid-year but had service use after turning 18 in 2015.

d. 11 individuals were missing deprivation status.

The 1-year prevalence rate of bipolar disorder treated in specialist services was 1.56 (95% CI 1.50–1.63) per 100 000 women and 1.20 (95% CI 1.14–1.26) per 100 000 men. Treated prevalence was 30% higher for women than men (RR = 1.30, 95% CI 1.22–1.39).

The majority (68%) had a recorded diagnosis of bipolar I disorder with or without other bipolar diagnoses (men 70%, women 66.8%). Bipolar II disorder was recorded among 14.3% of women compared with 9.6% of men, and although the *P*-value in Table 1 refers to the overall distribution across the prioritised bipolar diagnosis groups, the difference largely lies in bipolar I disorder and bipolar II disorder. In terms of psychiatric diagnoses (open/current during study year), around two-thirds had no other specific comorbid diagnoses recorded. A single comorbid condition was recorded for about one-quarter of people (22.2% of women, 24.4% of men) and a minority had two or more comorbidities (11.2% of women; 12.8% of men). Psychoses and alcohol and substance use were the most commonly recorded psychiatric comorbidities for men, and anxiety and mood disorders were most common for women (Table 1).

Table 2 shows the proportions of those identified with bipolar disorder accessing particular service types, and the annual mean rates (intensity of contact) of each type of service use by gender. Among the population accessing secondary mental health services with a diagnosis of bipolar disorder, one-third had a hospital admission in 2015, with women less likely to have had an admission than men (RR = 0.85, 95% CI 0.76–0.96), and having a lower mean rate of annual admissions. Almost all (92%) men and women were seen by community services at some time over the year, with a similar mean rate of contact of nearly two appointments per month (mean rate of just over 20 community treatment contacts per person per year for both men and women).

**Table 2:** Proportions of participants using different types of services, and rates of service use per person, by gender and service type

Variable	Women (*n* = 2121)		Men (*n* = 1518)		Rate ratio, women/men (95% CI)
	*n*	Proportion/mean number per year (95% CI)	*n*	Proportion/mean number per year (95% CI)	
In-patient admissions					
Any admission in year, proportion	644	30.4 (28.4–32.4)	541	35.6 (33.2–38.1)	0.85 (0.76–0.96)
Total number of admissions, mean per 100 patients per annum	1044	49.2 (46.3–52.3)	830	54.7 (51.1–58.5)	0.90 (0.82–0.99)
Community treatment					
Any treatment in year, %	1959	92.4 (91.1–93.5)	1390	91.6 (90.1–92.9)	1.01 (0.94–1.08)
Total number of treatments, mean per person per annum	47 293	22.3 (22.1–22.5)	33 493	22.1 (21.8–22.3)	1.01 (1.00–1.02)
Substance abuse treatment					
Any treatment in year, %	155	7.3 (6.2–8.5)	159	10.5 (9.0–12.1)	0.70 (0.56–0.87)
Total number of treatments, mean per person per annum	1809	0.9 (0.8–0.9)	2082	1.4 (1.3–1.4)	0.62 (0.58–0.66)
Forensic service treatment					
Any treatment in year, %	38	1.8 (1.3–2.5)	99	6.5 (5.3–7.9)	0.27 (0.19–0.40)
Total number of treatments, mean per person per annum	293	0.1 (0.1–0.2)	1106	0.7 (0.7–0.8)	0.19 (0.17–0.22)
Crisis treatment					
Any treatment in year, %	624	29.4 (27.5–31.4)	453	29.8 (27.5–32.2)	0.99 (0.87–1.11)
Total number of treatments, mean per person per annum	2043	1.0 (0.9–1.0)	1236	0.8 (0.8–0.9)	1.18 (1.10–1.27)
Support contacts					
Any contact in year, %	717	33.8 (31.8–35.9)	529	34.8 (32.4–37.3)	0.97 (0.87–1.09)
Total number of contacts, mean per person per annum	27 204	12.8 (12.7–13.0)	22 321	14.7 (14.5–14.9)	0.87 (0.86–0.89)

Substance use services were accessed by 7% of women and 11% of men in the study sample over 2015, and women had a lower mean number of contacts with these services than men (RR = 0.62, 95% CI 0.58–0.66). A small percentage were seen by forensic services (7% of men and 2% of women), with women also having a lower mean number of service contact than men (Table 2).

In contrast crisis treatment was accessed by 30% of men and 29% of women in the sample, but women had a higher mean number of crisis contacts (RR = 1.18, 95% CI 1.10–1.27). One-third of men and women with bipolar disorder had non-clinical support contacts recorded over the year, with a slightly lower mean number of such contacts for women (RR = 0.87, 95% CI 0.86–0.89).

Table 3 compares levels of assessment and treatment under the Mental Health Act by gender. Both assessment and treatment under the Mental Health Act were more common for men than women. In total, 19% of women and 24% of men were assessed for compulsory treatment over the year, and 16% of women and 22% of men received compulsory treatment over the same period, predominantly in the form of CTOs.

**Table 3:** Treatment under the Mental Health Act

Variable	Women (*n* = 2121)		Men (*n* = 1518)		Rate ratio, women/men (95% CI)
	*n*	% (95% CI)	*n*	% (95% CI)	
Assessment orders in year					
Any, %	406	19.1 (17.5–20.9)	371	24.4 (22.3–26.7)	0.78 (0.68–0.90)
Treatment orders in year					
Any, %	333	15.7 (14.2–17.3)	340	22.4 (20.3–24.6)	0.70 (0.60–0.82)
Any community treatment order in year, %	294	13.9 (12.4–15.4)	305	20.1 (18.1–22.2)	0.69 (0.59–0.81)
Any in-patient, %	160	7.5 (6.5–8.8)	165	10.9 (9.3–12.5)	0.69 (0.56–0.86)

## Discussion

### Main findings

This study has identified a complete national population of adults with a current diagnosis of bipolar disorder receiving specialist mental healthcare over a single year. It is important to note that this represents only those receiving specialist mental healthcare and is not representative of bipolar disorder in the community as a whole. However, it does represent every such case across the entire country. Women made up 58% of patients accessing secondary mental healthcare for bipolar disorder. When considered as a proportion of the general population, women were 30% more likely to receive specialist care for bipolar disorder.

Only one-third of the cohort had comorbid diagnoses recorded, and these varied by gender. Two-thirds of the cohort received only out-patient treatment over the 1-year period, and the average level of contact with community services was nearly two contacts per month over that year. Overall, women received less intensive treatment, with lower rates of high intensity treatment (in-patient treatment, compulsory treatment and forensic treatment) and a lower rate of support contacts than men. A similar proportion of women and men accessed services in crisis but women who saw crisis services were seen more times than men.

### Treatment numbers

The total number of people identified as receiving treatment for bipolar disorder in our study was 3639, which is considerably lower than the 12-month prevalence of bipolar disorder in New Zealand, previously reported from a community-based sample as 1% (0.6% bipolar I disorder; 0.4% bipolar II disorder).^3^ Our study also identified a higher proportion of people with bipolar I disorder compared with bipolar II disorder (of those with a specific diagnosis, 85% had a bipolar I disorder diagnosis). The discrepancy between numbers identified as having bipolar disorder in the national prevalence survey and those identified through mental health services is likely to be related to low rates of treatment contact for people with bipolar disorder, particularly bipolar II disorder in New Zealand. A large community study in New Zealand found that 12.2% of people with bipolar disorder had treatment contact in the year of onset, and only 53.2% eventually made treatment contact with a median duration of delay of 13 years.^3^ Bipolar I disorder is also more likely to result in contact with specialist mental health services than bipolar II disorder, and more than two-thirds of our sample had a bipolar I disorder diagnosis.

### Interpretation of our findings and comparison with findings from other studies

In our study more women than men in secondary mental health services had a current diagnosis of bipolar disorder, despite evidence of a similar prevalence by gender from the population survey.^3^ This may reflect the higher use of mental health services, particularly out-patient treatment, by women generally^32–34^ or that women are more likely to remain engaged with services regardless of diagnosis. These New Zealand findings on gender balance are compatible with the findings of an Austrian study that found that women made up 60% of those admitted to hospital for bipolar disorder, and a Danish study that found that women made up 54% of people in first contact with mental health services for bipolar disorder.^17,20^

It is likely that rates of comorbidity are underestimated in this study because of underrecording of psychiatric diagnoses within the PRIMHD system. Diagnoses in PRIMHD are based on coding from clinical records. These are not based on systematic interviews such as the Mini International Neuropsychiatric Schedule or Structured Clinical Examination for DSM-IV but on clinical interviews that may not seek or record symptoms of other conditions once a diagnosis of bipolar disorder has been made. Of course the presence of comorbid conditions may have important implications for treatment and prognosis.^35,36^ The fact that they have not been recorded at a rate that could be expected may suggest that they have not been elicited and may have important clinical implications and may suggest the need to raise awareness regarding comorbidity among clinicians treating bipolar disorder.

The men in our study had higher levels of recorded comorbid psychosis, and higher rates of hospital admissions, substance abuse treatment and forensic treatment. The higher rates of hospital admission suggests that men may be more acutely unwell when they present for treatment, in line with another New Zealand study suggesting that men were more likely than women to present to services in a manic phase.^37^ The higher rates of psychoses and hospital admission may also be because men are more likely to have comorbid substance use disorder, which is associated with higher rates of manic psychosis and consequently higher rates of admission.^36^ The higher rates of forensic service admissions for men may be reflective of the higher rates of arrest for men with psychotic disorders^38^ and criminal activity.^15^ The higher rates of substance use disorder treatment in our study is probably reflective of the higher rates of comorbid substance use in men in general and in this sample.^17,37^

The women in our study had higher rates of recorded comorbid anxiety. Some other clinical and epidemiological studies have found this pattern of higher rates of comorbid anxiety among women with bipolar disorder compared with men, although others have found similar rates of anxiety for both men and women with bipolar disorder.^39^ It is possible that in the context of a clinical interview rather than a systematic research interview women are more likely to report symptoms than men. Comorbid generalised anxiety disorder (the most common comorbid anxiety disorder in bipolar disorder) is associated with more severe bipolar disorder course and increased suicidality.^40^ As noted for general comorbidity, the rates of recorded anxiety disorder found in our study are much lower than expected^39^ once again related to the method of eliciting and recording symptoms.

The rates of compulsory hospital admission under the Mental Health Act were similar (approximately 27% of those with at least one admission) to reported rates for bipolar disorder in Austria (25% of admitted patients).^20^ National data shows that men are 1.6 times more likely to be subject to compulsory treatment in New Zealand mental health services for all psychiatric diagnoses.^41^ This matched with our study, where males were similarly more likely to be subject to compulsory treatment orders to a similar magnitude (RR = 1.43 for men relative to women – results in tables presented for women relative to men). This difference was similar across CTOs and in-patient orders. In contrast, a Swiss study found that involuntary admissions for bipolar disorder did not differ by gender.^42^ However in the Swiss study patients had to consent to participation, introducing significant bias into the recruitment process.

There is some evidence that people with bipolar disorder experience a significant delay in diagnosis and treatment of bipolar disorder after initiation of specialist mental healthcare (median diagnostic delay 62 days and median treatment delay 31 days), particularly for those who have prior diagnoses of substance use disorder.^43^ This may in part account for the higher rates of compulsory treatment for men who also have higher rates of substance use.

### Strengths and limitations

This study provides a national census of public specialist treatment for bipolar disorder, and enables the examination of treatment by gender including treatment in community settings. Because of the very low levels of private psychiatric care in New Zealand,^44^ this is likely to represent the vast majority of those who are accessing specialist psychiatric care for bipolar disorder. However, it does not include those receiving non-specialist treatment in primary care or not receiving formal treatment.

There are some limitations inherent in the use of the PRIMHD data. First, as noted, it only includes patients who have contact with specialist services and therefore only examines this group and not the wider group of all patients with bipolar disorder. Second some patients who have contact with services for less than 3 months will not have a diagnosis recorded.^45^ Third, coding is for a particular episode of care that usually, but not always, reflects the longitudinal course of the illness. This can result in apparent comorbid diagnoses that are actually part of the primary diagnosis – for example psychosis within a bipolar I disorder. Fourth, comorbid diagnoses are not always recorded because they are not enquired about or recorded in clinical files. The prevalence estimates for psychiatric comorbidities in this study should be interpreted with caution, and in many cases will represent an underestimate of the true prevalence in this patient group. However, it is probable that the underreporting was similar across gender so that although the rates reported may not reflect the true rates the differences are likely to be valid.

In conclusion, although bipolar disorder is equally common in men and women in the population, women are more likely to be in contact with specialist services for bipolar disorder. The patterns of comorbid diagnosis and treatment received varied by gender. Women were more likely to receive out-patient treatment only and have recorded comorbid anxiety, whereas more men had recorded substance use disorder, were convicted of crimes when unwell, received compulsory treatment and received in-patient treatment.

## Data Availability

The data that support the findings of this study are available from the Ministry of Health, New Zealand. Restrictions apply to the availability of these data, which were used under license for this study

## Supplementary material

For supplementary material accompanying this paper visit http://dx.doi.org/10.1192/bjo.2020.117. click here to view supplementary material
