# Supplementary material for: Gender and mental health service use in bipolar disorder: national cohort study
Source: BJPsych Open. 2020 Nov 6;6(6):e138. doi: 10.1192/bjo.2020.117 (PMC7745236; doi:10.1192/bjo.2020.117)
Supplement: Supplementary file 1 [file S2056472420001179sup001.docx]

Supplementary Table 1 Comorbid Psychiatric Diagnoses

| **Diagnosis Category** | **Specific diagnoses** | **ICD10** | **DSM IV** |
| --- | --- | --- | --- |
| Psychoses | Schizophrenia, Schizoaffective disorder, other psychotic disorder | F20-F25, F28-29 | 2951-57, 2959, 297-8 |
| Alcohol related disorder incl abuse/dependence |  | F10 | 291x, 3030, 3039, 3050 |
| Substance related disorder inc abuse/dependence (excluding nicotine) |  | F11-F16, F18-F19 | 292x, 304x, 3052-3059 |
| Depression and other mood disorders | Major Depressive Disorder, other mood disorder | F320-F323, F328, F329, F33x, F34-F39 | 2962-2963, 311, 2969 |
| Anxiety and related | Anxiety disorder, OCD, Adjustment disorder, PTSD | F40-F45, F48 | 3000-3008, 3090-3099 |
| Personality disorder |  | F60-69 | 3010-3019 |
